# Anti-Spoilage Activity and Exopolysaccharides Production by Selected Lactic Acid Bacteria

**DOI:** 10.3390/foods11131914

**Published:** 2022-06-27

**Authors:** Giovanna Iosca, Luciana De Vero, Giulia Di Rocco, Giancarlo Perrone, Maria Gullo, Andrea Pulvirenti

**Affiliations:** 1 Department of Life Sciences, University of Modena and Reggio Emilia, 42122 Reggio Emilia, Italy; giovanna.iosca@unimore.it (G.I.); giulia.dirocco@unimore.it (G.D.R.); maria.gullo@unimore.it (M.G.); andrea.pulvirenti@unimore.it (A.P.); 2 Institute of Sciences of Food Production (ISPA), National Research Council (CNR), Via G. Amendola 122/O, 70126 Bari, Italy; giancarlo.perrone@ispa.cnr.it; 3 Interdepartmental Research Centre for the Improvement of Agri-Food Biological Resources (BIOGEST-SITEIA), University of Modena and Reggio Emilia, 42124 Reggio Emilia, Italy

**Keywords:** sourdough, antimould activity, antibacterial activity, food safety, exopolysaccharides, starter culture

## Abstract

In this study, eight lactic acid bacteria (LAB) strains, previously isolated from traditional and gluten-free sourdoughs, and selected for their potential in improving the sensory and rheological quality of bakery products, were screened against some common spoilage agents. The anti-mould activity was tested using strains of the species *Fusarium graminearum*, *Aspergillus flavus*, *Penicillium paneum* and *Aspergillus niger.* Regarding the antibacterial activity, it was assessed against four strains of the species *Escherichia coli*, *Campylobacter jejuni*, *Salmonella typhimurium* and *Listeria monocytogenes*. Furthermore, LAB strains were evaluated for their ability to produce exopolysaccharides, which are gaining considerable attention for their functional properties and applicability in different food industrial applications. A strain-specific behaviour against the moulds was observed. In particular, *F. graminearum* ITEM 5356 was completely inhibited by all the LAB strains. Regarding the antibacterial activity, the strains *Leuconostoc citreum* UMCC 3011, *Lactiplantibacillus plantarum* UMCC 2996, and *Pediococcus pentosaceus* UMCC 3010 showed wide activity against the tested pathogens. Moreover, all the LAB strains were able to produce exopolysaccharides, which were preliminarily characterized. The assessed features of the LAB strains allow us to consider them as promising candidates for single or multiple starter cultures for food fermentation processes.

## 1. Introduction

Lactic acid bacteria (LAB) have been used, since ancient times, to preserve a wide range of fermented foods, including bread, vegetables, and dairy products.

Several LAB strains can produce different kinds of metabolites, including active amino acids, peptides, sugars, and volatile compounds, that contribute also to the final flavour of fermented food products [1]. Moreover, some LAB have beneficial technological applications in fermented foods and beverages due to their functional activity [2].

Many researchers have focused on the health-promoting effects of LAB-driven fermentation, including probiotic, immunomodulatory, anti-hypertensive, anti-oxidant, anti-cholesterolemic, anti-inflammatory, and anti-diabetic effects, among others [3,4,5].

Recently, LAB have been investigated as preservative agents to control spoilage agents, which are commonly present in cereals and cereal-derived food products, causing quality and sensory deterioration [6]. Although chemical preservatives are still widely used, the food industry is forced to provide innovative products by reconsidering the microbial fermentation process and bio-compounds as an alternative to satisfy the consumer’s demands for healthy and clean-label food [7].

Therefore, the research is pushed to obtain new starter cultures able to drive the fermentation process and enhance the microbiological safety and quality of foods.

Different LAB strains are known to produce antifungal compounds (including organic acids, peptides and fatty acids) able to control moulds, such as those belonging to *Aspergillus*, *Penicillium* and *Fusarium* genera, that usually cause bakery product deterioration and are dangerous for health [8,9,10].

Additionally, several LAB may inhibit the growth of spoilage and common pathogenic bacteria such as *Escherichia coli*, *Salmonella* spp., *Staphylococcus* spp. and *Campylobacter* spp., thanks to a synergistic effect of the pH decrease and production of different metabolites (e.g., lactic acid, acetic acid, bacteriocins and bacteriocins-like compounds) [11,12].

Above their antifungal and antibacterial activities, LAB are also able to produce exopolysaccharides (EPSs), which can strongly contribute to improving the shelf-life and palatability of food products. Generally, EPSs are synthesized and excreted by a variety of microorganisms, including LAB and acetic acid bacteria [13,14,15], and are classified into homo-EPS (HoEPS), such as glucans and fructans, composed of one repeated monosaccharide unit, and hetero-EPS (HeEPS), which are composed of several repeating units of sugars, such as pentose, hexose, or uronic acids, and may be branched or unbranched [16,17]. The use of EPSs in food production, as a natural alternative to commercial food additives, offers many advantages resulting in desirable rheological changes in foods, such as increased viscosity, reduced syneresis, and improved texture [18]. EPSs can act as emulsifying, thickening, and stabilising agents, providing firmness, creaminess, and mouthfeel, which are particularly desired for the improvement of gluten-free products [1,19]. Other positive attributes are related to their prebiotic, antitumor, immunomodulatory, and blood cholesterol-lowering activity [18,20,21].

In recent studies, the EPSs antagonistic roles to microbial pathogens, including viruses, bacteria and fungi, have also been investigated, although there is not a wide knowledge of the mechanisms responsible for this activity [13,22].

Since both the anti-spoilage activity and the EPSs production are features influenced by several factors, such as microbial growth and metabolic activity of each strain, microbial interaction, as well as the type of carbohydrates and enzymes [23,24], the screening of different LAB strains is a fundamental step for the design of ad hoc industrial starter.

Accordingly, culture collections are fundamental tools for innovative research and scientific services to customers and stakeholders as they can screen in-house the interesting candidates to select the best one with the specific features.

In this study, eight LAB strains of the Unimore Microbial Culture Collection (UMCC) were tested for their antimould and antimicrobial activity, as well as the production of EPSs. These strains were previously identified and characterised for different aroma compound production [25] and their applicability in gluten-free fermented products [26]. The final purpose was to consider their potential use as innovative starter cultures able to enhance the organoleptic properties, texture, palatability, and safety of different food matrices.

## 2. Material and Methods

### 2.1. Lactic Acid Bacteria Strains

The LAB strains used in this work (Table 1) are maintained in UMCC by cryopreservation at −80 °C, in MRS broth (Oxoid, Milan, Italy) and 25% (*v/v*) glycerol (Carlo Erba, Milan, Italy). LAB strains were grown overnight in MRS broth at 30 °C before being used for the anti-spoilage screenings or the EPSs production.

### 2.2. Antimould Activity Screening

The LAB strains were tested for their antimould activity using a dual agar plate assay, according to Hassan et al. [27]. Four moulds provided by the Agro-Food Microbial Culture Collection (ITEM Collection, www.ispa.cnr.it/Collection, accessed on 24 June 2022) of the Institute of Sciences of Food Production, CNR (Bari, Italy), specifically *Fusarium graminearum* ITEM 5356, *Aspergillus flavus* ITEM 7828, *Aspergillus niger* ITEM 7090, and *Penicillium paneum* ITEM 13081, were used for the screening. Briefly, LAB strains were grown overnight in MRS broth at 30 °C, and 100 μL of each culture at 10^8^ cfu/mL was mixed with 10 mL of MRS agar. Plates were incubated at 30 °C for 24 h and then overlaid with 10 mL of Potato Dextrose agar (PDA, Oxoid, Milan, Italy). The inoculum for testing antimould activity was prepared from spores of each mould, collected after 5 days of growing the cultures on PDA. 10 µL of spore suspension at 10^6^ cfu/mL was placed in the middle of the PDA plates. All plates were incubated in aerobic conditions at 25 °C for 9 days and the radial inhibition of fungal growth was measured [28]. The experiment was repeated three times with three replicates each time.

### 2.3. Antibacterial Activity Screening

The antibacterial activity of LAB strains was assessed against four bacterial pathogens: *Campylobacter jejuni* ATCC 33250, *Escherichia coli* ATCC 43888, *Listeria monocytogenes* ATCC 19115, and *Salmonella typhimurium* ATCC 14028. The agar diffusion assay method was applied to evaluate the diameters of inhibition zones following the protocol of Arrioja-Bretón et al. [29]. Briefly, a layer of 15 mL of MRS agar was poured into the Petri dishes and, after solidification, overlaid by 10 mL of Brain Heart Infusion agar (BHIA, Oxoid, Milan, Italy). A total of 100 µL of each actively growing pathogen strain (10^6^ cfu/mL) was streaked onto BHIA. Then, 20 µL of each LAB strain at 10^8^ cfu/mL were loaded into wells of 8 mm in diameter and depth, up to the underlying MRS medium, obtained with the help of a borer. For each plate, two wells were set. The diameter of the inhibition zone was measured after cultivation for 48 h at 30 °C. The experiment was repeated five times with two replicates each time.

### 2.4. Screening for EPSs Production

A preliminary screening was performed to observe the ropy phenotype typical of the EPS-producing LAB [30]. In detail, active cultures of the LAB strains were streaked onto MRS-sucrose agar, prepared by supplementing MRS medium with 60 g/L of sucrose. The high sugar concentration was applied to produce a nutrient stress factor and increase the production of EPSs [31]. Cultures were incubated at 30 °C for 4 days. Subsequently, LAB exhibiting slimy surfaces and a ropy condition after touching the colony with a loop were reported as promising EPS-producing strains and transferred to the MRS-sucrose broth for the extraction procedure.

#### 2.4.1. EPS Extraction Procedure

The extraction of EPSs from the selected LAB was performed by adapting the protocol reported by Wang et al. [32]. Specifically, 1 mL of each strain was used to inoculate flasks with 500 mL of MRS-sucrose broth. The flasks were incubated at 30 °C for 4 days and then heated at 100 °C for 30 min. The supernatant was recovered after centrifugation at 6000 rpm for 30 min and EPSs were precipitated overnight at 4 °C by addition of an equal volume of cold absolute ethanol. After, EPSs were recovered by centrifugation (6000 rpm for 120 min at 4 °C) and the pellet was redissolved in 10 mL of distilled water with gentle heating. Neutral sugars were eliminated by dialysis for 48 h, then all the samples were lyophilized for 48 h. To purify the samples from proteins, treatment with trichloroacetic acid (TCA) 14% (stirred overnight at room temperature) was performed. The proteins were removed by centrifugation at 6000 rpm for 30 min, and EPSs were precipitated using an equal volume of ethanol at −21 °C. The pellet was dissolved in double-distilled water and lyophilized again for 48 h. The final samples obtained were weighed and the values were expressed in g/L.

#### 2.4.2. EPSs Characterization

To describe and evaluate the composition of the EPSs obtained, all the freeze-dried samples were characterised by attenuated total reflectance–Fourier transform infrared (ATR-FTIR) spectroscopy and liquid chromatography tandem mass (LC-MS/MS) spectrometry.

##### ATR-FTIR Spectroscopy

Some crystals of extracted EPSs were loaded on the ATR-FTIR. Spectra were collected using a Perkin Elmer Spectrum TWO UTR-FTIR with PerkinElmer^®^ Spectrum IR software, version 10.6.0.893. To improve the signal-to-noise ratio for each spectrum, 32 interferograms with a spectral resolution of ±4 cm^−1^ were averaged. Background spectra, which were collected under identical conditions, were subtracted from the sample spectra.

##### ESI-ION TRAP LC MS/MS (LCMSIT) Analysis

The EPSs samples were separated on a microscale Accucore TM HILIC column (2.6 μm; 50 mm × 2.1 mm; Thermo Fisher, Milan, Italy) using an LC-MS(n) Ion Trap 6310 A (Agilent Technologies, Milan, Italy). Solvent A consisted of H_2_O with formic acid 0.1%; solvent B was acetonitrile. The following gradient conditions were used: t 0–3 min, 90% solvent B; t 3–6 min, 80% solvent B; t 6–10 min, 50% solvent B; t 10–15 min, 50% solvent B, and back to 90% solvent B until stop time of 25 min. A flow of 0.4 mL/min was applied [33]. For sample injection, the EPSs (6 mg/mL) were resuspended in H_2_O. The samples were centrifuged for 1 min at 13,000 rpm before analysis to remove any particles from the sample solution. Aliquots of 15 μL were injected using an autosampler.

### 2.5. Statistical Analysis

Statistical analysis of data was determined by one-way analysis of variance (ANOVA), followed by Tukey’s test, using SPSS 20.0 software (SPSS, Inc., Chicago, IL, USA); *p*-values ≤ 0.05 were considered statistically significant. All measurements were performed at least in triplicates. The results were expressed as mean ± standard deviation.

## 3. Results and Discussion

### 3.1. Assessment of the Antimould Activity

**Figure 1:**
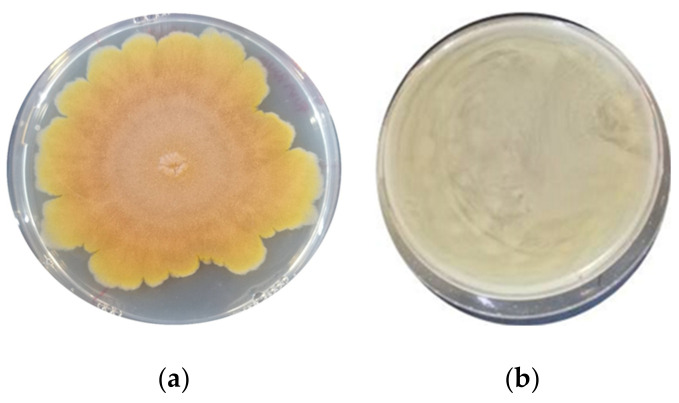
*Fusarium graminearum* on control plate (**a**) and on plate inoculated with *Leuconostoc citreum* UMCC 3011 (**b**).

**Figure 2:**
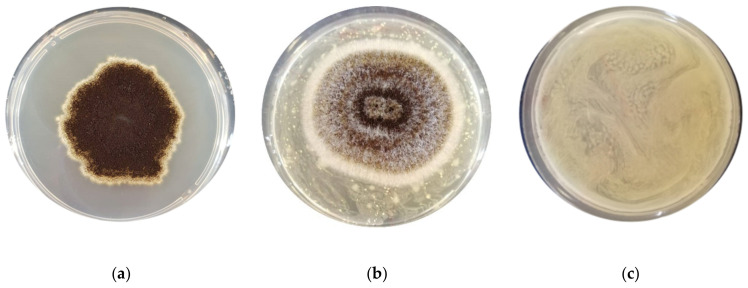
*Aspergillus niger* on control plate (**a**) and on plate inoculated with *Leuconostoc citreum* UMCC 2993 (**b**) and UMCC 3011 (**c**).

The antimould activity of the LAB strains was assessed after nine days of incubation at the optimal growth temperature (25 °C) for the moulds (Table 2). A different strain behaviour was observed for each specific mould. *F. graminearum* ITEM 5356 and *A. flavus* ITEM 7828 were the most sensitive moulds, followed by *A*. *niger* ITEM 7090. Specifically, the *F. graminearum* strain was completely inhibited by all the LAB strains tested (Figure 1), while the *A*. *flavus* strain was inhibited only by *Lacp. plantarum* UMCC 2996 and UMCC 3009, *Furl*. *rossiae* UMCC 3002, *Lenl. parabuchneri* UMCC 2992, and *Fl. sanfranciscensis* UMCC 2990. Against the same mould, a slighter inhibition was detected by *Leuc. citreum* (UMCC 3011 and UMCC 2993) and *P. pentosaceus* UMCC 3010. In contrast, no inhibition was observed against *P*. *paneum* ITEM 13081. Regarding *A. niger*, a complete inhibition was reported with the strains of *P*. *pentosaceus*, *Furl*. *rossiae*, *Lenl. parabuchneri*, and *Leuc. citreum* (UMCC 3011). A distinctive antimould activity was observed by *Leuc. citreum* UMCC 3011 and UMCC 2993, previously isolated from Panettone doughs (Figure 2). This diverse activity could be correlated with the different origin of the two strains, since the Panettone doughs contained a different amount of sugars and fat, which affects the environmental conditions, such as the osmotic pressure and pH, causing different stress responses [25].

Among the various LAB tested by several authors, those belonging to *P. pentosaceus* and *Lacp. plantarum* were reported to be the most prominent strains as bio-preservatives able to produce different compounds active against filamentous fungi [8,9,34,35]. In addition, other LAB species such as *Leuc. citreum* and *Furl. rossiae*, isolated from Italian *durum* wheat semolina, revealed a strong activity against *A. niger* [36]. Obligatory and facultative heterofermentative LAB such as *Lacp*. *plantarum*, *Fl. sanfranciscensis*, *Furl. rossiae*, *Leuc. citreum,* and *Lenl. parabuchneri* seem to display major antimould activity compared to the homofermentative bacteria such as *Lactobacillus delbrueckii* subsp. *Lactis*, *Lacticaseibacillus paracasei,* and *P. pentosaceus* [8]. Beyond lactic and acetic acid, LAB produce several metabolites which have antifungal activity, such as formic acid, propionic acid, butyric acid, n-valeric acid, hexanoic acid, diacetyl, hydrogen peroxide, caproic acid, 3-hydroxy fatty acids, phenyllactic and 4-hydroxy-phenyllactic acids, compounds of proteinaceous nature, and reuterin matrices [37,38,39,40,41].

### 3.2. Assessment of the Antibacterial Activity

**Figure 3:**
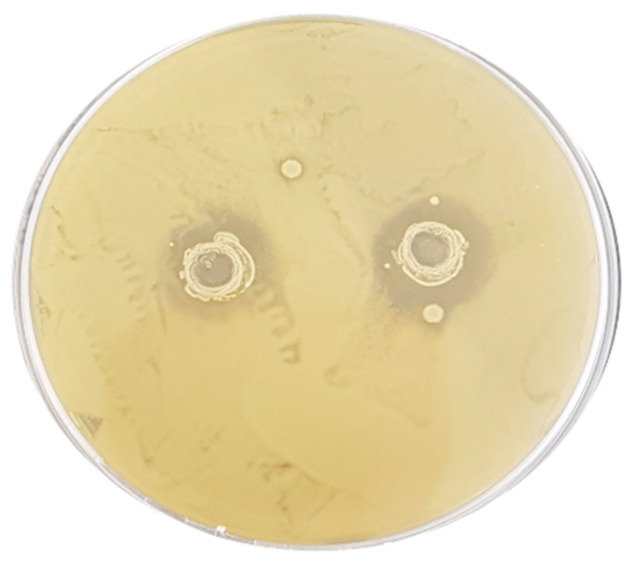
Inhibition zones due to the activity of *Leuconostoc citreum* UMCC 3011 against *Escherichia coli* ATCC 43888, observed by a dual plate agar assay.

To evaluate the antibacterial capacity of the LAB strains, a well diffusion assay was performed and the diameter inhibition zone (DIZ), expressed in mm, was measured after 48 h of incubation. The strains *Furl. rossiae* UMCC 3002, *Lenl. parabuchneri* UMCC 2992, and *Fl. sanfranciscensis* UMCC 2990 did not show activity against the selected pathogens, and consequently no inhibition zone was detected (Table 3). Regarding the other five strains, great variability of the inhibitory action was observed. The strains *Leuc. citreum* UMCC 3011, *Lacp*. *plantarum* UMCC 2996, and *P. pentosaceus* UMCC 3010 showed wide antimicrobial activity on all the pathogens. In particular, the strain *Leuc. citreum* UMCC 3011 was effective on all the pathogen strains tested, with non-significant differences. Its activity on *E. coli* ATCC 43888 is shown in Figure 3. The highest activity was detected in the case of the *P. pentosaceus* strain against *L. monocytogenes* (DIZ 18.00 ± 0.16) and *C. jejuni* (DIZ 15.6 ± 0.44).

On the contrary, *Leuc*. *citreum* UMCC 2993 demonstrated antimicrobial activity only on strains *C. jejuni* (DIZ 8.2 ± 0.25 mm) and *L. monocytogenes* (DIZ 10.70 ± 0.14 mm), respectively. Regarding the strains *Lacp. plantarum* UMCC 3009, no activity was observed on *S*. *typhimurium* ATCC 14028. The different behaviour of LAB strains, belonging to the same species but isolated from different samples, highlights that the antimicrobial activity is a strain-specific trait, as already observed by Divyrashee et al. [42].

The antibacterial activity of LAB has been studied during the last years by using different strains isolated from different substrates [43,44]. Strains of *Pediococcus* were reported to exhibit strong inhibitory ability against pathogenic bacteria, along with useful features such as high tolerance to sodium chloride, bile salt, and low pH [43]. Other studies have described the efficient antibacterial activity of *Leuc. citreum* and *Leuc. mesenteroides* strains against spoiling bacteria, including *L. monocytogenes*, in meat [45] and fruit [46]. The inhibitory capacity of LAB is generally ascribed to the final products of their fermentative metabolism. Organic acids, especially lactic and acetic acids, formic acid, free fatty acids, ammonia, ethanol, hydrogen peroxide, diacetyl, acetoin, 2,3-butanediol, acetaldehyde, benzoate, bacteriolytic enzymes, and bacteriocins, are the main compounds involved in the inhibitory activity against various spoilage bacteria [47].

However, as recently stated, the number of individual compounds produced by LAB is often insufficient to sustain the inhibitory phenotype, which most likely arises from the synergistic or additive bioactivity of a combination of compounds [48].

### 3.3. Characterization of the EPSs Produced by LAB Strains

The preliminary screening of the eight LAB on the MRS-sucrose plates, by picking-up colonies grown with an inoculation loop, allowed observation of the formation of filaments; thus, all the tested strains were considered as potential EPS-producers.

The EPSs extraction protocol included several steps for the removal of protein content, small neutral sugars, and salts. The pure EPSs obtained showed a spongy texture of about 1.4 g/L, which was a good amount considering that generally the EPSs production of different LAB strains is reported to vary between 0.01 g/L and 0.4 g/L under non-optimized culture conditions, and can reach multiple of this amount under ideal conditions [18,49]. In our tested condition, the high amount of sugar (60 g/L of sucrose) may have contributed to the increased production of EPSs. The possible explanations for the increased EPS synthesis under the stress of high sugar concentration include osmosis, the unlimited supply of sugar building blocks, and high energy availability [31,49]. Generally, the EPSs yield produced by LAB mainly depends on several parameters, such as temperature, pH, oxygen tension, incubation period, and microbial growth conditions [50].

#### 3.3.1. ATR-FTIR Analysis

The LAB’s ability of producing EPSs was firstly tested by ATR-FTIR, exploiting the method recently used to detect active groups of hydroxyls, carboxylics, and carbonyls [51], and identify chitin oligomers from enzymatically digested samples [52,53,54].

**Figure 4:**
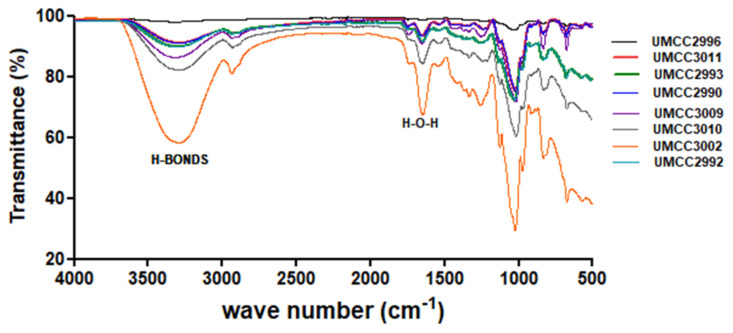
ATR-FTIR spectra of the freeze-dried samples obtained from the tested lactic acid bacteria strains.

All the samples had similar spectra, in which the presence of polysaccharides was evident (Figure 4). The IR spectra of sugars from the literature were used for comparison.

The two different regions of the spectrum analysed are the ‘sugar fingerprint’ region (1500–950 cm^−1^), which is fundamental for the identification and the structural characterization of polysaccharides, and the region of the functional groups (2500–3600 cm^−1^), which gives information on the crystallinity of the sample (Figure 4). In particular, the first region contains bands assigned to sugar ring C–C and C–O vibration modes (1104 cm^−1^ and 1030 cm^−1^, respectively) in the case of cellulose, but they could vary by about 20–30 cm^−1^ depending on the different kind of sugars, as well as on the antisymmetric stretching of the C1–O–C4 moiety of the glycosidic linkage (1160 cm^−1^). The bands between 3400 cm^−1^ and 3270 cm^−1^ are attributed to O–H and stretching of the C=O----H–O moieties for the intra-sheet hydrogen bond in crystalline polymer, together with the bands at 700 cm^−1^ (out-of-plane vibrations of O–H groups or rotational vibrations of the whole water molecule), 1640 cm^−1^ (H–O–H angle vibrations), and 2100 cm^−1^ (vibrations from the scission and rocking of water) [53,54,55]. The differences in the intensities and shape in the bands at 3300 and 1670 cm^−1^ are due to the content of water within the sample and the lengths of the polymers [53].

To note, the sample related to *Furl*. *rossiae* UMCC 3002 (orange line in Figure 4) displays very intense OH signals indicative of a considerable amount of water entrapped in the sample, leading to a less crystalline shape. For instance, oligomers consisting of fewer monomer units (5–7 glucose units) are more hydrophilic, and this is consistent with the broadening of the band at 3300 cm^−1^. Finally, the different intensity could also depend on the number of oligomers in the samples, but we were able to recognise EPSs in all the samples. Moreover, considering that in chito-oligosaccharides there are bands at 1637 and 1545 cm^−1^ that can therefore be assigned to the acetyl groups of chitin, in particular to C=O and N–H vibrations, respectively, the absence of these bands indicates that no acetyl amidate EPSs are present in the blend [56].

#### 3.3.2. LCMSIT Analysis

**Figure 5:**
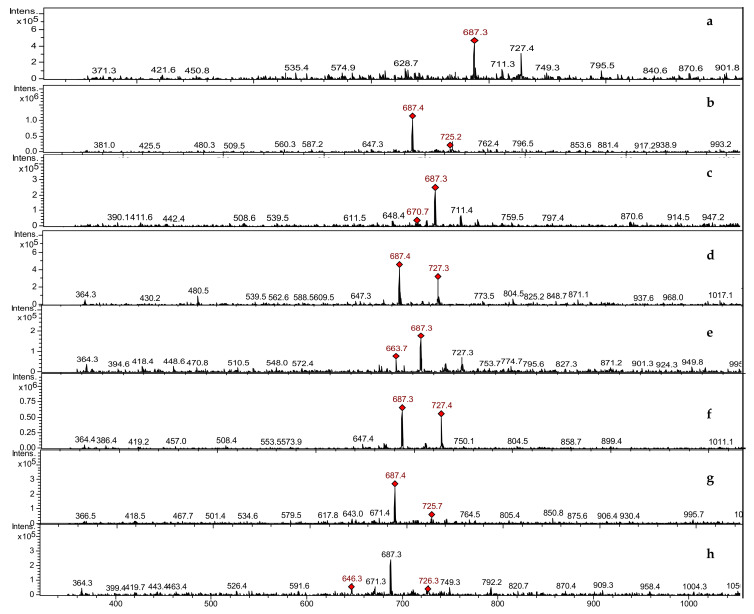
MS spectra recorded in positive mode for the samples obtained after EPSs extraction from the eight lactic acid bacteria strains tested: *Fructilactobacillus sanfranciscensis* UMCC 2990 (**a**), *Lentilactobacillus parabuchneri* UMCC 2992 (**b**), *Leuconostoc citreum* UMCC 2993 (**c**), *Lactiplantibacillus plantarum* UMCC 2996 (**d**), *Furfurilactobacillus rossiae* UMCC 3002 (**e**), *Lactiplantibacillus plantarum* UMCC 3009 (**f**), *Pediococcus pentosaceus* UMCC 3010 (**g**), *Leuconostoc citreum* UMCC 3011 (**h**). Highlighted in red are the masses that have been subsequently fragmented for MS/MS analysis.

The LCMSIT analysis was performed to further assess the presence of EPSs and preliminarily identify their composition. By calculating the *m/z* ratios of oligomers of different length and monomer combinations, and by searching in the MS spectra of eluted peaks of all samples, it was possible to confirm the presence of EPSs (Figure 5).

It is known that the majority of LAB are able to synthesise different types of EPSs, producing oligomers of glucose, galactose, dextran, levan, rhamnose, arabinose, and mannose [17,57,58]. These oligomers are composed of repeating units of glucose (180.06 g/mol) and rhamnose (164.06 g/mol).

**Figure 6:**
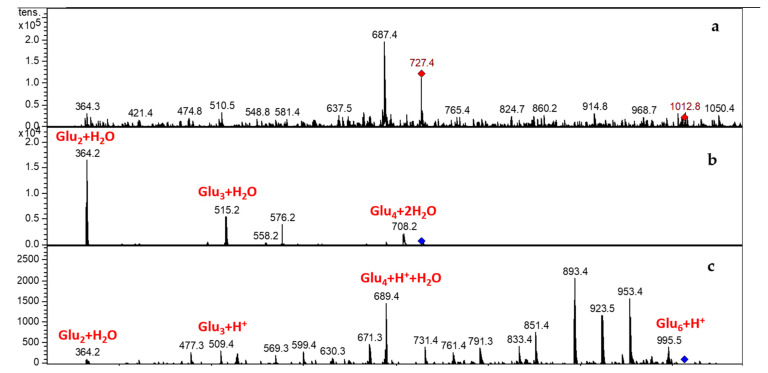
MS spectrum (**a**) and MS/MS spectra of the peak at 727.4 m/z (**b**) and 1012.8 m/z (**c**) of the sample obtained from *Leuconostoc citreum* UMCC 2993. Highlighted in red are the masses corresponding to the loss of a glucose unit.

The most abundant MS signals for all samples fall at 687.4 m/z and at 727.2 m/z, which correspond to the mass of a tetra-glucose (666.24 g/mol) with one or two water molecules, or a sodium atom coordinated, with or without a water molecule. The details for the sample obtained from *Leuc*. *citreum* UMCC 2993 are reported in Figure 6, which includes the MS spectrum (in the first row), together with two MS/MS spectra of the peak at 727.4 m/z (tetra-glucose + 2H_2_O) and the peak at 1012.8 m/z, respectively. The identified masses are highlighted in red in the figure. Similar data were observed by the MS/MS analysis of all the other samples (Figures S1–S7).

The EPSs detection and characterization have shown an abundant production of glucose oligomers, along with levans, dextrans, and rhamnose oligomers. These oligomers are different in structure, but their homo-polymeric or hetero-polymeric character cannot be achieved by a simple MS or a tandem approach. Nevertheless, our analyses unambiguously ascribe the EPSs products to the glucans and fructans families. Generally, the production of HoEPS from LAB is markedly higher than that of HeEPS. This is because the former does not require active transport steps and energy consumption, except for the biosynthesis of the extracellular enzymes [17,59].

A complete EPS composition could be achieved by combining different analytical methods to detail monosaccharide composition, ring conformation, linkage, degree of branching, and molecular weight [17].

## 4. Conclusions

Spoilage agents represent a severe threat to food industries, driving them to use chemical preservatives to control the contaminations. However, consumers are increasingly attracted by natural and healthy food, rejecting more elaborated products containing chemicals. Therefore, the selection of LAB strains with high efficacy against spoilage agents is of great interest for building up innovative starter cultures able to produce high quality and safe products with also appreciated flavours, which satisfy the new market demand.

The eight UMCC LAB, screened in this study, demonstrated antimould and antibacterial activity, even if a strain-specific behaviour was observed. In particular, *F*. *graminearum* ITEM 5356 was completely inhibited by all the LAB strains. Regarding the antibacterial activity, the strains *Leuc. citreum* UMCC 3011, *Lacp*. *plantarum* UMCC 2996 and *P*. *pentosaceus* UMCC 3010 showed wide activity against the tested pathogens. Moreover, all the LAB strains were able to produce EPSs, which were preliminarily ascribed to the family of glucans and fructans. The assessed features of the LAB strains tested, enriched also by their ability to produce different organic acids and aromas compounds, as stated in our previous work, allow us to consider them as promising candidates to be used as single or multiple starter cultures for food fermentation processes and clean-label products.

## Figures and Tables

**Table 1:** Lactic acid bacteria strains from Unimore Microbial Culture Collection (UMCC) tested in the present study.

		Species	Origin	
UMCC ID	Other Denomination		Isolation Source	Region
UMCC 2990	BMA8	*Fructilactobacillus sanfranciscensis*	sourdough type I	Emilia Romagna (Italy)
UMCC 2992	BIA2	*Lentilactobacillus parabuchneri*	dough for Panettone	Emilia Romagna (Italy)
UMCC 2993	BIA6	*Leuconostoc citreum*	dough for Panettone	Emilia Romagna (Italy)
UMCC 2996	BFA1	*Lactiplantibacillus plantarum*	dough for Panettone	Emilia Romagna (Italy)
UMCC 3002	BIB7	*Furfurilactobacillus rossiae*	dough for Panettone	Emilia Romagna (Italy)
UMCC 3011	BFB3	*Leuconostoc citreum*	dough for Panettone	Emilia Romagna (Italy)
UMCC 3009	LC1RC13	*Lactiplantibacillus plantarum*	gluten-free sourdough	Sicily (Italy)
UMCC 3010	LL1C9	*Pediococcus pentosaceus*	gluten-free sourdough	Sicily (Italy)

Data source: UMCC Database, https://umcc.bio-aware.com/ (accessed on 26 May 2022).

**Table 2:** Activity of eight lactic acid bacteria strains against the tested moulds.

MOULDS	CONTROL	*Leuc. citreum* UMCC 3011	*Lacp. plantarum* UMCC 2996	*P. pentosaceus* UMCC 3010	*Lacp. plantarum* UMCC 3009	*Leuc. citreum* UMCC 2993	*Lenl. parabuchneri* UMCC 2992	*Fl. sanfranciscensis* UMCC 2990	*Furl. rossiae* UMCC 3002
*F. graminearum* ITEM 5356	32.2 ± 0.66 ^b^	0 ± 0 ^a^	0 ± 0 ^a^	0 ± 0 ^a^	0 ± 0 ^a^	0 ± 0 ^a^	0 ± 0 ^a^	0 ± 0 ^a^	0 ± 0 ^a^
*A. flavus* ITEM 7828	41.8 ± 0.32 ^c^	35.7 ± 0.38 ^b^	0 ± 0 ^a^	27.1 ± 0.93 ^b^	0 ± 0 ^a^	34.5 ± 0.34 ^bc^	0 ± 0 ^a^	0 ± 0 ^a^	0 ± 0 ^a^
*P. paneum* ITEM 13081	38.5± 0.53 ^ab^	35.5 ± 0.23 ^ab^	39.4 ± 0.47 ^b^	38.2 ± 0.21 ^ab^	39.4 ± 0.13 ^b^	37.0 ± 0.37 ^ab^	34.1 ± 0.19 ^a^	34.1 ± 0.19 ^a^	33.5 ± 0.40 ^a^
*A. niger* ITEM 7090	31.8 ± 1.03 ^b^	0 ± 0 ^a^	33.2 ± 0.66 ^bc^	0 ± 0 ^a^	32.1 ± 0.53 ^b^	33.1 ± 0.38 ^bc^	0± 0 ^a^	39.4± 0.30 ^c^	0 ± 0 ^a^

The radial growth (in mm) of the moulds was detected after 9 days of incubation. Superscript letters represent the statistical differences within a row. Different letters are significantly different (*p* < 0.05). Data are presented as mean ± SD, *n* = 9.

**Table 3:** Antimicrobial activity showed by some of the lactic acid bacteria strains tested in this study.

PATHOGEN STRAIN	*Leuc. citreum* UMCC 3011	*Lacp. plantarum* UMCC 2996	*P. pentosaceus* UMCC 3010	*Lacp. plantarum* UMCC 3009	*Leuc. citreum* UMCC 2993	*Lenl. parabuchneri* UMCC 2992	*Fl. sanfranciscensis* UMCC 2990	*Furl. rossiae* UMCC 3002
*E. coli* ATCC 43888	12.6 ± 0.17 ^Ad^	7.2 ± 0.27 ^Ac^	11.3 ± 0.24 ^Ad^	4.4 ± 0.16 ^Bb^	0 ± 0 ^Aa^	0 ± 0 ^Aa^	0 ± 0 ^Aa^	0 ± 0 ^Aa^
*S. typhimurium* ATCC 14028	13.4 ± 0.43 ^Ab^	13.4 ± 0.43 ^Bb^	11.3 ± 0.60 ^Ab^	0 ± 0 ^Aa^	0 ± 0 ^Aa^	0 ± 0 ^Aa^	0 ± 0 ^Aa^	0 ± 0 ^Aa^
*L. monocytogenes* ATCC 19115	12.3 ± 0.09 ^Aa^	11.8 ± 0.14 ^Ba^	18.0 ± 0.16 ^Bb^	10.9 ± 0.22 ^Ca^	10.7 ± 0.14 ^Ca^	0 ± 0 ^Aa^	0 ± 0 ^Aa^	0 ± 0 ^Aa^
*C. jejuni* ATCC 33250	13.1 ± 0.21 ^Ab^	14.6 ± 0.34 ^Bb^	15.6 ± 0.44 ^ABb^	11.6 ± 0.45 ^Cab^	8.2 ± 0.25 ^Ba^	0 ± 0 ^Aa^	0 ± 0 ^Aa^	0 ± 0 ^Aa^

Diameter inhibition zone (DIZ) is expressed in mm. ^a–c^ Mean values with different letters are significantly different (*p* < 0.05). Capital letters represent the differences within a column, while small letters represent differences within a row. Data are presented as mean ± SD, *n* = 10.

## Data Availability

All related data and methods are presented in this paper. Additional inquiries should be addressed to the corresponding author.

## Supplementary Materials

The following are available online at https://www.mdpi.com/article/10.3390/foods11131914/s1, Figure S1: MS spectrum (**a**) and MS/MS spectrum (**b**) for the sample obtained from *Fructilactobacillus sanfranciscensis* UMCC 2990; Figure S2: MS spectrum (**a**) and MS/MS spectrum (**b**,**c**) for the sample obtained from *Lentilactobacillus parabuchneri* UMCC 2992; Figure S3: MS spectrum (**a**) and MS/MS spectrum (**b**,**c**) for the sample obtained from *Lentiplantibacillus plantarum* UMCC 2996; Figure S4: MS spectrum (**a**) and MS/MS spectrum (**b**,**c**) for the sample obtained from *Furfurilactobacillus rossiae* UMCC 3002; Figure S5: MS spectrum (**a**) and MS/MS spectrum (**b**,**c**) for the sample obtained from *Lactiplantibacillus plantarum* UMCC 3009; Figure S6: MS spectrum (**a**) and MS/MS spectrum (**b**,**c**) for the sample obtained from *Pediococcus pentosaceus* UMCC 3010; Figure S7: MS spectrum (**a**) and MS/MS spectrum (**b**) for the sample obtained from *Leuconostoc citreum* UMCC 3011.
