# Supplementary material for: Anti-Spoilage Activity and Exopolysaccharides Production by Selected Lactic Acid Bacteria
Source: Foods. 2022 Jun 27;11(13):1914. doi: 10.3390/foods11131914 (PMC9265762; doi:10.3390/foods11131914)
Supplement: Supplementary file 1 [file foods-11-01914-s001.zip › foods-1772423-supplementary.pdf]

Supplementary material

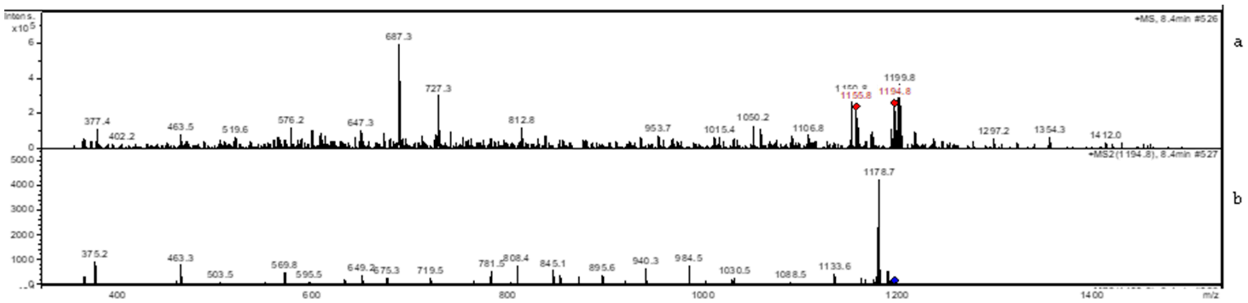

Figure S1. MS spectrum (**a**) and MS/MS spectrum (**b**) for the sample obtained from *Fructilactobacillus sanfranciscensis* UMCC 2990. In red are highlighted the masses of 1155.8 m/z corresponding to $[Glu_7+H^+]^+$ and of 1194.8 m/z for $[Glu_7+2H_2O+H^+]^+$ and in blue are the molecular ions subjected to a second fragmentation.

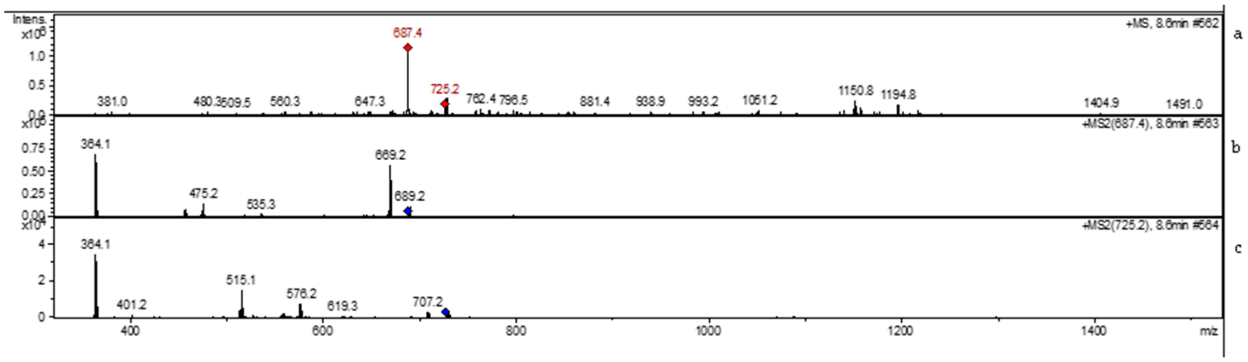

Figure S2. MS spectrum (**a**) and MS/MS spectrum (**b**,**c**) for the sample obtained from *Lentilactobacillus parabuchneri* UMCC 2992. In red are highlighted the masses of 687.4 m/z corresponding to $[Glu_4+H_2O+H^+]^+$ and of 725.2 m/z for $[Glu_4+2H_2O+Na^+]^+$ in blue are the molecular ions subjected to a second fragmentation.

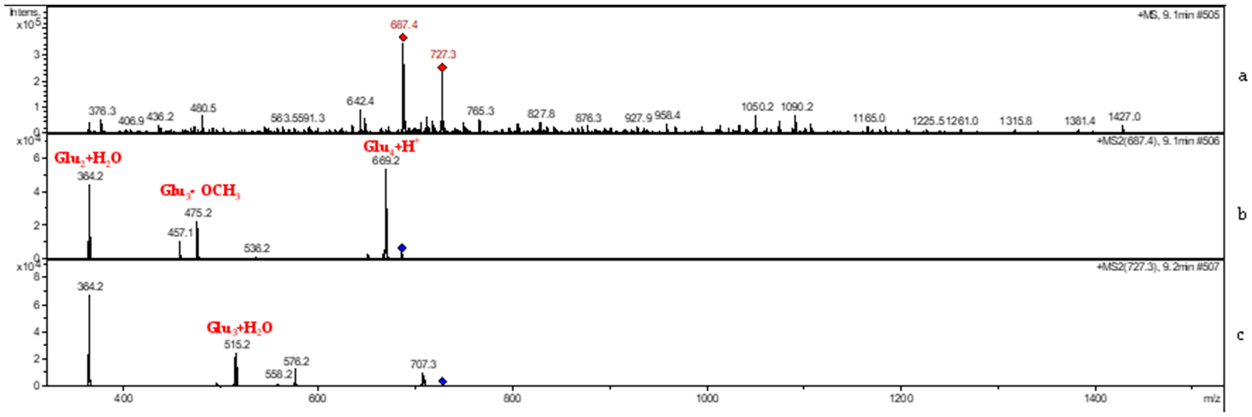

Figure S3. MS spectrum (**a**) and MS/MS spectrum (**b**,**c**) for the sample obtained from *Lentiplantibacillus plantarum* UMCC 2996. In red are highlighted the masses of 669.2 m/z corresponding to $[Glu_4+ H^+]^+$ and of 727.3 m/z for $[Glu_4+2H_2O+Na^+]^+$ in blue are the molecular ions subjected to a second fragmentation.

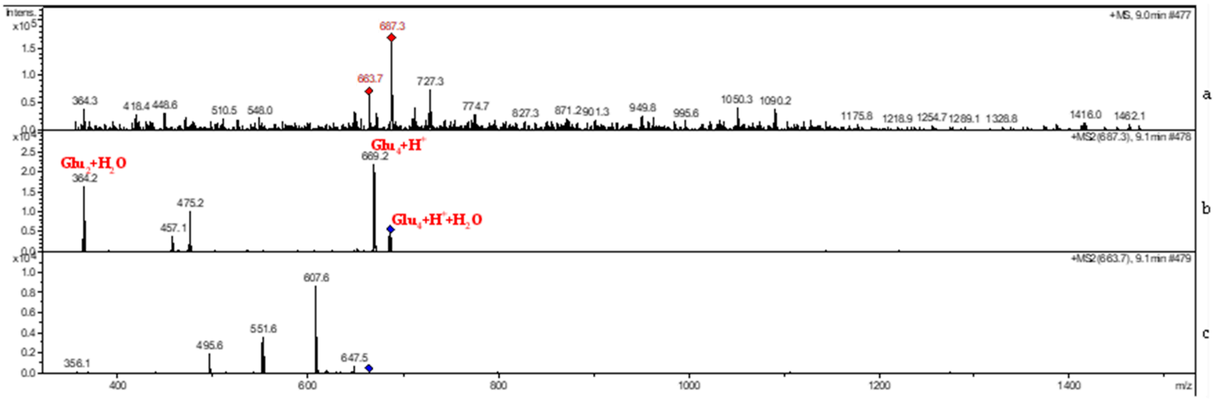

Figure S4. MS spectrum (**a**) and MS/MS spectrum (**b**,**c**) for the sample obtained from *Furfurilactobacillus rossiae* UMCC 3002. In red are highlighted the masses of 663.7 m/z corresponding to a probable contaminant and of 687.3 m/z for $[Glu_4+H_2O+H^+]^+$ in blue are the molecular ions subjected to a second fragmentation.

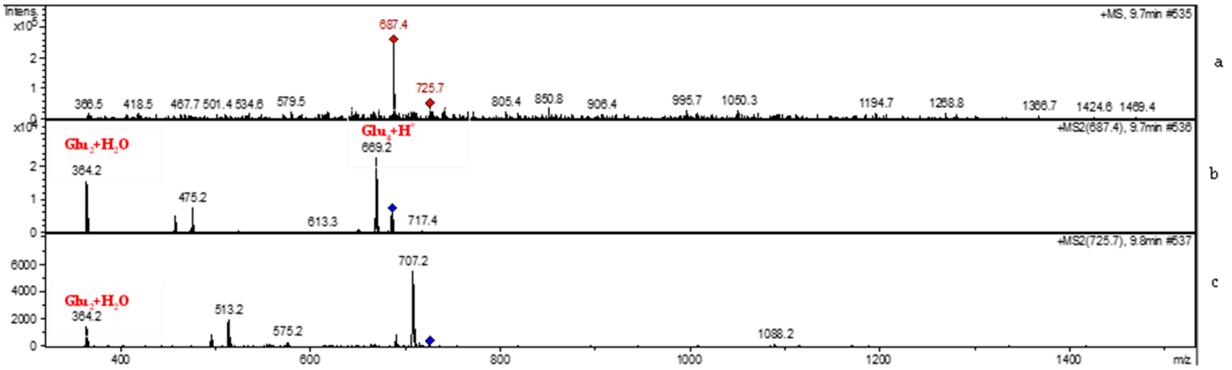

Figure S5. MS spectrum (**a**) and MS/MS spectrum (**b**,**c**) for the sample obtained from *Lactiplantibacillus plantarum* UMCC 3009. In red are highlighted the masses of 669.2 m/z corresponding to $[Glu_4+ H^+]^+$ and of 725.7 m/z for $[Glu_4+2H_2O+Na^+]^+$ in blue are the molecular ions subjected to a second fragmentation.

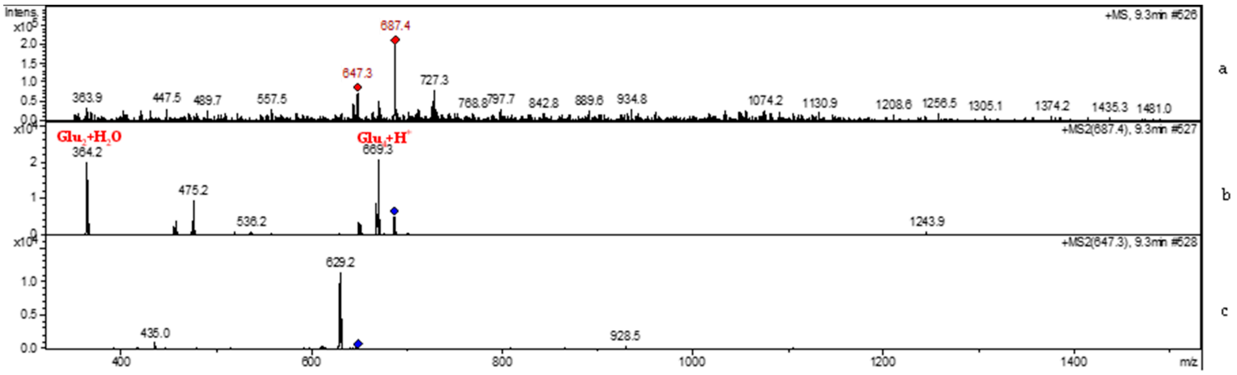

Figure S6. MS spectrum (**a**) and MS/MS spectrum (**b**,**c**) for the sample obtained from *Pediococcus pentosaceus* UMCC 3010. In red are highlighted the masses of 647.3.2 m/z corresponding to $[Man_4+ H_2O + Na^+]^+$ and of 687.4 m/z for $[Glu_4+2H_2O+H^+]^+$ in blue are the molecular ions subjected to a second fragmentation.

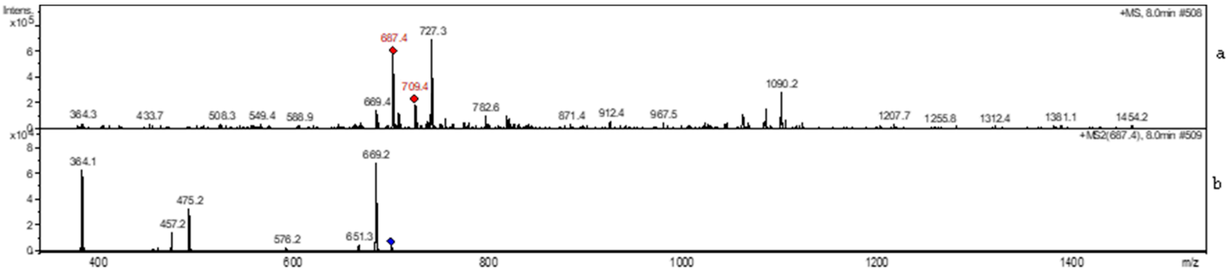

Figure S7. MS spectrum (**a**) and MS/MS spectrum (**b**) for the sample obtained from *Leuconostoc citreum* UMCC 3011. In red are highlighted the masses of 687.4 m/z corresponding to $[Glu_4+H_2O+H^+]^+$ and of 709.3 m/z for $[Glu_4+2H_2O+H^+]^+$ in blue are the molecular ions subjected to a second fragmentation.
